# Infection control practice in countries with limited resources

**DOI:** 10.1186/1476-0711-10-36

**Published:** 2011-10-22

**Authors:** Emine Alp, Hakan Leblebicioglu, Mehmet Doganay, Andreas Voss

**Affiliations:** 1 Department of Infectious Diseases and Clinical Microbiology, Faculty of Medicine, Erciyes University, Kayseri, Turkey; 2 Department of Infectious Diseases and Clinical Microbiology, Faculty of Medicine, Ondokuz Mayis University, Samsun,Turkey; 3 Department of Clinical Microbiology, Radboud University Nijmegen Medical Centre, Nijmegen, The Netherlands

**Keywords:** Infection control, developing country, limited resource, multi-drug resistant pathogen

## Abstract

Nosocomial infections and their control are a world-wide challenge. The prevalence of nosocomial infections is generally higher in developing countries with limited resources than industrialized countries. In this paper we aimed to further explain the differences with regard to infection control challenges between Turkey, a country with "limited" resources, and the Netherlands, a country with "reasonable" resources. Infrastructure of hospitals, low compliance of hand hygiene, understaffing, overcrowding, heavy workload, misuse of personal protective equipments, late establishment of infection control programme are major problems in limited-resources countries. These problems cause high infection rates and spread of multi-drug resistant pathogens. To improve the control and prevention of infections in countries with limited resources, a multi-facet approach is needed.

## Review

Nosocomial infections and their control are a world-wide challenge. Next to the raised morbidity and mortality of patients, nosocomial infections furthermore increase the costs of healthcare due to added antimicrobial treatment and prolonged hospitalization. Since the prevalence of nosocomial infections is generally higher in developing countries with limited resources (> 40%) [1], the socio-economic burden is even more severe in these countries. Adding the lack of infection control to the above-mentioned problems, may explain, why nosocomial infections deepen a downward healthcare spiral. While problem micro-organisms such as methicillin-resistant *Staphylococcus aureus *(MRSA) and multi-drug resistant (MDR) *Acinetobacter*, or nosocomial infections, such as bloodstream infections (BSI) and ventilator-associated pneumonia (VAP), are shared by all countries independent of their resources, the extend of the prevalence of MDR micro-organisms, and the frequency of nosocomial infections clearly differs due to inadequate infection control [2-4].

Aim of this paper is to further explain the differences with regard to infection control challenges between Turkey, a country with "limited" resources, and the Netherlands, a country with "reasonable" resources.

Nosocomial infections constitute an important health-care problem in Turkey, especially in university hospitals. The in hospital prevalence of healthcare-associated infections was reported to be 13.4% [3], reaching 48.7% in intensive care units [4]. In comparison the prevalence of nosocomial infections in the Netherlands (according to the national surveillance system PREZIES) is 7.2%, ranging from 6.7% to 9.0% in non-academic and academic settings, respectively [5]. The infrastructure of most Turkish hospitals probably effects infection control practices and consequently raises the infection rates. Most of the hospital wards have few single rooms and are not generally equipped with specific isolation rooms. The lack of single and isolation rooms, missing control of temperature, humidity and air quality aid the multiplication and dissemination of MDR microorganisms. Furthermore, the uncomfortable conditions such as high temperatures during the summer months, may affect HCW's work performance. In addition wards, including intensive care units (ICU), are cramped with insufficient bed-to-bed space and very high turn-around time of patients. The bed-to-bed distance is at least 150 cm in ICUs in the Netherlands, whereas it is generally 100 cm in most of the hospitals in Turkey. Inadequate infrastructures and overcrowding hamper the application of infection control practices and thereby contribute to the dissemination of MDR microorganisms and increased occurrence of HAIs.

One of the most important infection control measures is hand hygiene. In Turkey, the compliance of health-care workers is generally poor (< 33%) [6-8]. The major reasons for non-compliance are heavy workload, inadequate structure (lack of sinks, difficult access to hygiene products), and behavioural aspects. All of the above is amplified by the combination of overcrowding and insufficient numbers of healthcare workers. In the Netherlands, on average the nurse/patient ratio in intensive care units is 1/1, whereas in Turkey this is usually 1/3-5. Furthermore, when cleaning their hands, Turkish HCWs still prefer hand washing over alcoholic hand-rubs, despite the fact that it is well known that hand disinfection at the point-of-care is much faster, more effective and allows for higher compliance, since it takes less time [9]. Unfortunately branded hand-rubs are relatively expensive for countries with "limited" resources and not easy to obtain for all hospitals. However, recently the Ministry of Health of Turkey began a hand hygiene campaign, called as "Danger In Your Hands", all around the country. This campaign includes several tools (education programmes, posters, distribution of alcoholic hand rubs and paper towels, etc.) to improve the hand hygiene compliance in healthcare settings. In the Netherlands, health-care structure (including sufficient amount of sinks and - in ICUs - bedside hand-rub dispensers) and resources would theoretically allow for 100% compliance with hand hygiene [9]. Still, a recent multi-centre study only showed an average hand hygiene compliance of 19%, even lower than the compliance observed in Turkey (V. Erasmus, G. Vos, personal communication). Still, other factors such as awareness of infection control problems, knowledge and individual behaviour, impact the prevention of nosocomial infections. In an interventional study, we compare the hand hygiene practice during catheter care in intensive care units in the Netherlands and in Turkey. Both ICUs have alcohol products at the bedside, education programmes and reminders, but the compliances with hand hygiene were significantly different (18% and 65% in Turkey and the Netherlands, respectively). After an educational intervention, the compliance did not change in the Turkish ICUs, whereas it increased in Dutch centre [10]. However, a recent study from Turkey showed that regular training for the residents in charge of inserting intravascular catheters and the nurses and interns who maintain the catheters is highly effective in reducing the rate of CRI in large teaching hospitals [11]. Multiple factors affect the behaviour of humans (family culture, knowledge, beliefs, personality features, role model, rewards, etc.) [12]. In countries with limited resources HCWs may lose their labour enthusiasm and stick to the minimum routine work, due to the low financial reward (salary). Since, most of the behavioural patterns of humans take shape in their childhood, hand hygiene promotion programmes should be carried out in the community to educate future HCWs.

The use of personal protective equipment (PPE) (mask, gloves, gowns, etc.) is a basic part of infection control. In the Netherlands all hospitals are well supplied with necessary PPEs. However there are problems with acquisition and adequate use of PPEs in Turkey. Most of the hospitals first got acquainted with respiratory masks after bird flu threat, when CDC recommended FFP3 masks. Today, many hospitals only have surgical and FFP3 masks, whereas in Dutch healthcare settings the much cheaper FFP1 and FFP2 masks are used, since there is no evidence that FFP3 masks are needed. Due to the discomfort when using FFP3 masks (certainly in models without a valve) the compliance is probably decreased. On the other hand, there is extravagance for some of the PPEs. Other situations in which scare resources are probably misspend are the use of gloves instead of hand hygiene and the use of shoe covers in ICUs and sometimes even for all visitors entering the hospital.

Despite the increasing problems with nosocomial infections and emergence of multi-drug resistant pathogens in Turkey, infection control programs have not been a national priority in the past. In the 1980's, 30 years after the start of infection control in the Netherlands [13,14], infection control programs were started and a limited number of hospitals set-up a national surveillance system (NOSO-line) in Turkey. Only in 2006 the surveillance system was extended to many hospitals by Refik Saydam Hygiene Center. All the hospitals in Turkey, report their hospital infection rates by this system and try to improve infection control practices according to these results. Also Turkey is the membership of the International Nosocomial Infection Control Consortium (INICC), which is the multinational research network established to control HAIs in hospitals in limited-resource countries [15,16]. And recently (2000) the Turkish Society of Hospital Infection and Control was formed and started to work on guidelines for national use and in 2005 the Ministry of Health reported statutes for infection control in hospitals. However the structure of the Dutch infection control centre (WIP) was long established at that time. Due to the above, Turkish HCWs are newly familiar with the principles of infection control measures.

Also due to the late establishment of infection control programs in Turkey, most of the hospitals in Turkey do not have sufficient number of infection control nurses, despite the fact that they are the mainstay of infection control programs. Only recently standardized training programmes for infection control nurses and doctors were implemented by the Ministry of Health in Turkey. Neither Turkey nor the Netherlands have a medical (sub-) specialty of infection control, but in both countries medical microbiologist and infectious diseases specialist lead the infection control teams.

High nosocomial infection rates in hospitals encourage physicians, especially surgeons, to use unnecessary and inappropriate antibiotics. In a multicenter point-prevalence study in Turkey, more than 50% of patients received inappropriate antimicrobial prescriptions [17]. In another study only 26% of the patients received appropriate antimicrobial prophylaxis [18]. Inappropriate antimicrobial use will further increase resistance-development in nosocomial pathogens. Due to the poor compliance with infection control measures and ineffective infection control programmes these microorganisms will spread within the healthcare settings. In most Turkish university hospitals, MRSA and multi-drug resistant *Acinetobacter *spp. are endemic, causing major outbreaks [19,20]. Furthermore, Gram-negative rods producing ESBLs and vancomycin-resistant enterococci (VRE) are emerging. Resistant strains can successfully be controlled by effective infection control program as shown with the control of MRSA by the "Search and Destroy" strategy used in the Netherlands since 1986, after introduction of MRSA into their healthcare system [13]. Early identification of carriers and "pre-emptive" isolation of patients on admission are the main stay of this infection control strategy. However, this policy is cumbersome and costly (surveillance, isolation) in countries with limited resources and/or high prevalence.

The use of molecular diagnostic and typing techniques to support infection control efforts has become routine in most developed countries. Yet many Turkish university hospitals do not have access to these techniques, thereby missing valuable data for outbreak investigations and rapid diagnostic tools to better control the spread of MDR-organisms.

## Conclusions

While increasing funds would obviously help to solve some of the above-mentioned problems, this is naturally not a solution for countries with limited resources. To improve the control and prevention of infections in countries with limited resources, a multi-facet approach is needed that is based on improved healthcare structures, increased knowledge, effective guidelines, behavioural changes, attitude adjustment, better and efficient use of existing resources, as well as international cooperation.

## Competing interests

The authors declare that they have no competing interests.

## Authors' contributions

EA and AV conceived and wrote the manuscript. HL and MD have been involved in revising the manuscript critically. All authors read and approved the final manuscript.
